# Laboratory Identification of Lupus Anticoagulant (LA) Using Different Activated Partial Thromboplastin Time (APTT) Assays

**DOI:** 10.1111/ijlh.14549

**Published:** 2025-08-27

**Authors:** Bárbara G. Barion, Bianca Stefanello, Maria Luiza S. A. de Paula, Thaís S. Saraiva, Paula R. Villaça, Vanderson Rocha, Fernanda A. Orsi, Tania R. F. da Rocha

**Affiliations:** ^1^ Hospital das Clínicas da Faculdade de Medicina de Sao Paulo Brazil; ^2^ University of Sao Paulo Medical School Brazil; ^3^ Department of Pathology, School of Medical Sciences University of Campinas (UNICAMP) Campinas Brazil

**Keywords:** antiphospholipid antibodies, antiphospholipid syndrome, APTT, lupus anticoagulant

## Abstract

**Introduction:**

The International Society of Thrombosis and Hemostasis (ISTH) guidelines suggest a three‐step evaluation for the detection of lupus anticoagulant (LA), including screening, mixing, and confirmation. According to the guidelines, the LA assay based on activated partial thromboplastin time (APTT) should include an initial screening step followed by a confirmatory step that uses a higher concentration of phospholipids in either bilayer or hexagonal form. For the activator, the guidelines recommend using silica, though ellagic acid is also an option. In this context, HemosIL Silica Clotting Time (SCT, Instrumentation Laboratory) is the only assay that fully complies with the guidelines. However, there are other assays available using different reagents, such as Dade Actin FSL/FS (Siemens Healthcare Diagnostics) and PTT‐LA/Staclot LA (Diagnostica Stago), and the relevance of these differences in LA detection is not known.

**Methods:**

This study compared the performance of the three platforms.

**Results:**

Out of 136 samples, the majority were from females (82%) with a median age of 41 years (IQR 32–50); 44 (32%) had a history of thrombosis, and 28 (21%) were on anticoagulants. PTT‐LA/Staclot LA had the highest sensitivity (100%) and specificity (100%). There was an almost perfect agreement between PTT‐LA/Staclot LA and Dade Actin FSL/FS (kappa 0.812). HemosIL SCT sensitivity was 100% and the specificity was 74%, which was increased to 99% by increasing the phospholipid concentration of the screening step.

**Conclusion:**

We observed a good agreement between PTT‐LA/Staclot LA and Dade Actin FSL/FS, and fair to moderate agreement with HemosIL SCT, whose performance improved with increasing phospholipid concentration. These results demonstrate that all three assays are comparable for APTT‐LA detection.

## 1 Introduction

Antiphospholipid syndrome (APS) is an autoimmune disease characterized by arterial, venous, or microvascular thrombosis, or recurrent miscarriage, associated with the presence of antiphospholipid antibodies (aPL) [1]. The detection of aPL in clinical laboratories relies on solid‐phase immunoassays (anticardiolipin and beta‐2 glycoprotein I) and functional assays like lupus anticoagulant (LA). The guidelines for Lupus Anticoagulant/Phospholipid‐Dependent Antibodies Subcommittee of the Scientific and Standardisation Committee (SSC) of the International Society of Thrombosis and Haemostasis (ISTH) recommend at least two coagulation assays operating on two different principles for LA assays, being diluted Russell's viper venom time (dRVVT) and LA‐sensitive activated partial thromboplastin time (APTT‐LA) [2, 3]. It is recommended that both assays be performed for all patients, as the dRVVT is valued for its specificity and robustness, while the APTT‐LA is preferred for its sensitivity [4, 5, 6].

The guidelines also recommend a three‐step method for LA testing, including screening, mixing, and confirmatory steps [7]. The screening step must be performed with a reagent known for its sensitivity or responsiveness to LA, and typically containing a low concentration of phospholipids, while the confirmatory step is performed with an increased phospholipid concentration [7, 8, 9]. The results suggest a positive LA when a prolonged clotting time is detected in the screening test, followed by a shortened clotting time detected in the confirmatory step [10].

The SSC‐ISTH guidelines recommend silica as the preferred activator for APTT‐LA assays [7], but ellagic acid can also be used [11]. In addition, the guidelines recommend that the confirmatory step be performed by increasing the concentration of the same phospholipids used in the screening test (bilayer or hexagonal) [7, 8, 9].

Currently, several platforms and reagents are available for APTT‐LA assays. The primary differences between these platforms lie in the composition of their reagents, particularly the type of activator and the source of phospholipids. Although all platforms adhere to the three steps recommended by the SSC‐ISTH guidelines—screening, mixing, and confirmatory—the choice of activator and phospholipid source may influence assay accuracy. Furthermore, data comparing these platforms, which could support the decision‐making process when selecting a platform for clinical laboratories, are scarce. In this context, the aim of our study was to compare the performance of HemosIL SCT, Dade Actin FSL/FS, and PTT‐LA/Staclot LA, evaluating their sensitivity, specificity, and degree of agreement. In addition, in a post hoc analysis, we sought to develop a modified HemosIL SCT protocol in order to improve the assay performance.

## 2 Materials and Methods

### 2.1 Plasma Samples

A total of 136 plasma samples was obtained from two laboratories in the University of Sao Paulo Medical School–Hospital das Clínicas (HCFMUSP) complex: the Hemostasis Laboratory of the Hematology and Cell Therapy Service and the Hemostasis Laboratory of the Instituto do Coração Professor Euryclides de Jesus Zerbini (INCOR). The study included samples that were sent to the participating laboratories with the request to investigate the presence of LA. Samples were collected from February 2024 to June 2024.

Blood samples were collected in tubes containing 3.2% sodium citrate and centrifuged at 3000 × g for 20 min at room temperature (RT) to obtain platelet‐poor plasma (PPP), as previously described [12]. The PPP was then divided into three aliquots and stored at −80°C until further use. To compare the three platforms evaluated in this study, aliquots from the same sample were used, ensuring a maximum interval of 3 weeks between all analyses.

### 2.2 APTT‐LA Assays

The samples were tested for LA using PTT‐LA/Staclot LA (Diagnostica Stago) on the STA R Max analyzer (Diagnostica Stago, Gennevilliers, France) at INCOR, while at HCFMUSP, testing was performed using Dade Actin FSL/FS APTT (Siemens Healthineers, Marburg, Germany) on the CS‐2500 system (Sysmex UK, Milton Keynes, UK), and HemosIL SCT (HemosIL Silica Clotting Time, Instrumentation Laboratory) on the ACL TOP 350 analyzer (Instrumentation Laboratory, Naples, Italy).

The cutoff values used in this study followed the manufacturers' recommendations and were internally validated. For the Dade Actin FSL/FS APTT assay, a cutoff ratio of 1.20 was used for both the screening and confirmatory steps. For the PTT‐LA/Staclot LA assay, we used a ratio of 1.3 in the screening step and a difference of 8 s in the confirmatory step. For the HemosIL SCT assay, we used the recommended cutoff value of 1.16 for the normalized SCT Ratio (SCT screen ratio/SCT confirm ratio), and the cutoff ratios for the screen and confirm tests were established (99th percentile) using normal samples and set at 1.19 and 1.17, respectively.

The 2020 SSC‐ISTH guidelines recommendations were followed for all samples. Initially, a screening test was performed, and when prolonged clotting time was detected, mixing tests were conducted simultaneously with the confirmatory test. Mixing tests were performed in a 1:1 proportion with negative control plasma.

Although the methodologies of the three manufacturers are grounded in the same principles (APTT‐based), there are considerable differences in reagent composition. The characteristics of these reagents are shown in Table 1.

**TABLE 1:** Reagent characteristics.

Manufacturer	Reagent	Activator	Phospholipids
Diagnostica stago	PTT‐LA	Silica	Rabbit
	Staclot LA		Hexagonal
Siemens healthineers	Actin FSL	Ellagic acid	Soy and rabbit
	Actin FS		Soy
Instrumentation laboratory	HemosIL silica clotting time (SCT screen)	Sílica	Synthetic
	HemosIL silica clotting time (SCT confirm)		Synthetic

*Note:* A detailed account of the specific equipment and reagents utilized, accompanied by a comprehensive analysis of their individual compositions.

In order to assess the sensitivity and specificity of the test, we considered a sample to be a true positive if it was positive in at least two of the three platforms used in this study. Conversely, we defined a false positive result as a result that only tested positive in one of the platforms.

### 2.3 Modified HemosIL Silica Clotting Time Protocol

After the initial tests, in an attempt to increase the specificity of the HemosIL SCT assay, we raised the concentration of phospholipids in the screening assay by adding 100 μL of the confirmatory reagent to the HemosIL SCT screen instead of the 50 μL recommended by the manufacturer. A new cutoff value was established for the modified screening reagent based on 135 samples from healthy donors and was calculated at the 99th percentile. The modified HemosIL SCT cutoffs were 1.32 for the screening ratio and 1.47 for the normalized SCT. All subsequent assay steps were performed in accordance with the manufacturer's instructions. A total of 124 study samples were analyzed using the modified protocol, while 12 samples were excluded due to insufficient volume. The screen ratio, confirm ratio, and normalized SCT values obtained with the modified HemosIL SCT among the 135 control samples, along with the cutoff calculations, are detailed in the Table S1.

### 2.4 Statistical Analysis

Descriptive analysis was performed using frequency tables for categorical variables, and numerical variables were expressed as the mean and standard deviation (normally distributed variable) or median and interquartile range (nonnormally distributed). First, specificity and sensitivity were performed for each APTT‐LA assay. Next, the kappa coefficient was used to evaluate the agreement between the different assays. We used the scale proposed by Landis and Koch for the kappa index [13], that is, an index kappa from 0 to 0.20 is defined as slight, between 0.21 and 0.40 as fair, 0.41 and 0.60 as moderate, 0.61 and 0.80 as substantial, and above 0.81 as almost perfect.

Statistical analyses were performed with MedCalc software (MedCalc Software, Ostend, Belgium) and GraphPad Prism 8.0 (GraphPad Software, USA).

## 3 Results

A total of 136 independent samples were tested in the study. Most of them were female (*n* = 112, 82%) with a median age of 41 years (IQR 32–50) (Table 2). Forty‐four samples (32%) were from patients with a history of thrombosis, and 28 (21%) from patients using anticoagulants, the majority of whom (61%) were on direct oral anticoagulants (DOAC).

**TABLE 2:** Clinical information provided with the test request.

	Origin of the samples (*n* = 136)
Sex, *n* (%)	
Male	24 (18)
Female	112 (82)
Age (median, IQR)	
Male	46 (33–65)
Female	40 (31–49)
History of thrombosis, *n* (%)	
Yes	44 (32)
No	15 (11)
No clinical information	77 (57)
Anticoagulation, *n* (%)	
Yes	28 (21)
No	28 (21)
No clinical information	80 (59)
Type of anticoagulation, *n* (%)	
Warfarin	5 (18)
UFH	1 (3)
LMWH	5 (18)
DOAC	17 (61)

*Note:* Information on the origin of the samples, including gender, age, history of thrombosis, and use and type of anticoagulation.

Abbreviations: DOAC, direct oral anticoagulant; IQR, interquartile range 25% and 75%; LMWH, Low‐molecular‐weight heparin; *n*, absolute number of patients; UFH, Unfractionated Heparin.

### 3.1 Agreement Between Assay

Out of the total 136 samples analyzed across three reagents, the PTT‐LA/Staclot LA detected 8 (6%) positive samples, Dade Actin FSL/FS APTT detected 9 (7%) positive samples, and HemosIL SCT detected 25 (18%) positive samples.

An agreement between HemosIL SCT and PTT‐LA/Staclot LA results was observed in 119 samples (87.5%), including 8 positive and 111 negative results. Concordant results between PTT‐LA/Staclot LA and Dade Actin FSL/FS APTT were noted in 133 samples (97.8%), comprising 7 positive and 126 negative results. Finally, concordance between Dade Actin FSL/FS APTT and HemosIL SCT results was observed in 116 samples (85.3%), with 7 positive and 109 negative results.

Therefore, the agreement was almost perfect between PTT‐LA/Staclot LA and Dade Actin FSL/FS APTT, moderate between HemosIL SCT and PTT‐LA/Staclot LA, and fair between HemosIL SCT and Dade Actin FSL/FS APTT. The findings are presented in Table 3.

**TABLE 3:** Agreement between the assays (Kappa coefficient).

	Kappa	95% IC	Level of agreement
PTT‐LA/Staclot LA versus Dade Actin FSL/FS APTT	0.812	0.604–1.000	Almost perfect (97.8%)
HemosIL SCT versus PTT‐LA/Staclot LA	0.434	0.227–0.642	Moderate (87.5%)
Hemosil SCT versus Dade Actin FSL/FS APTT	0.348	0.139–0.558	Fair (85.3%)

*Note:* Results of the kappa coefficient test between the different reagents tested in the study.

### 3.2 Sensitivity and Specificity

**FIGURE 1:**
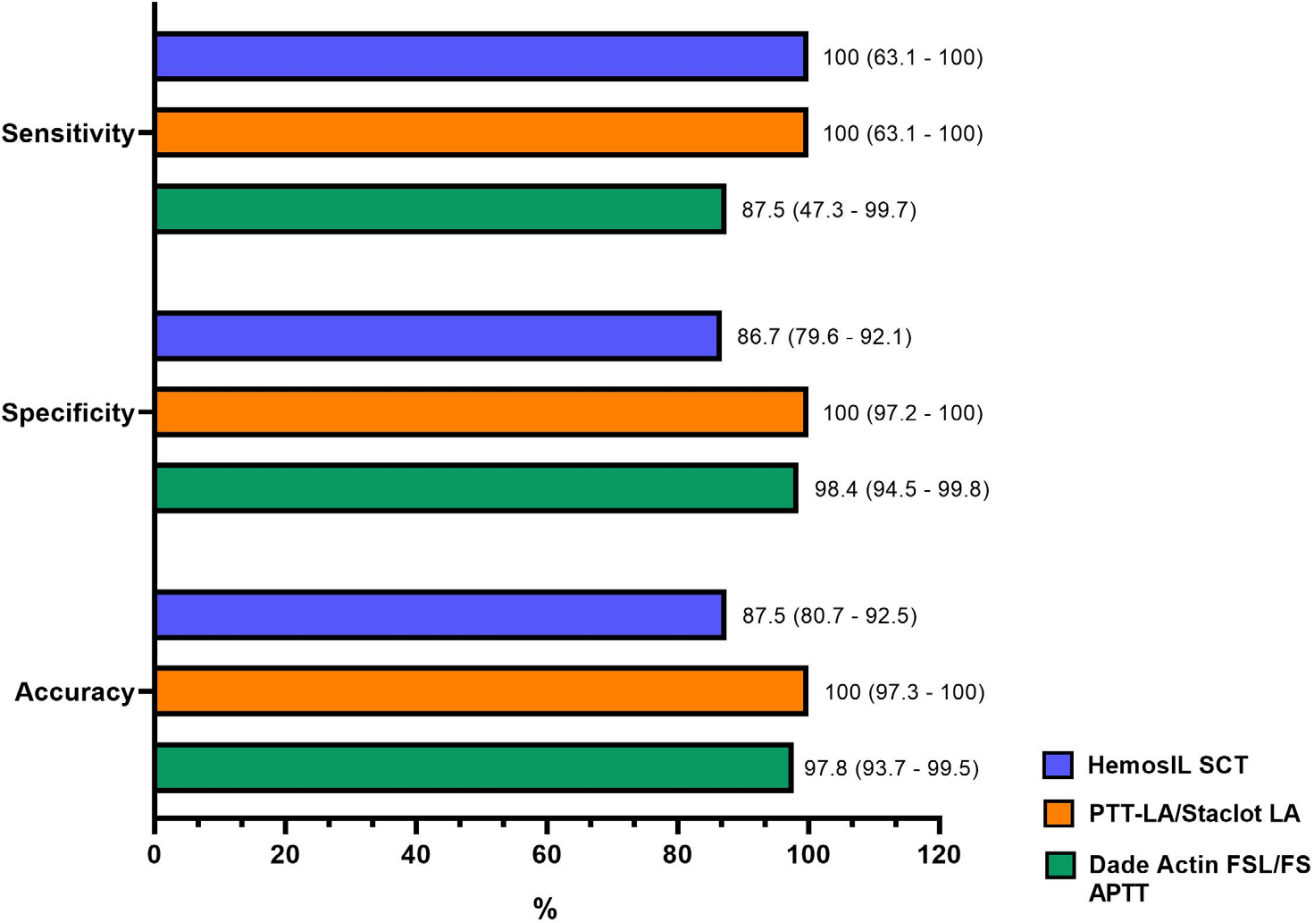
Sensitivity, specificity and accuracy of the reagents. This figure illustrates the sensitivity, specificity, and accuracy of three different platforms used in this study for detecting lupus anticoagulant: Hemosil SCT (blue), PTT‐LA/StaClot LA (orange), and Dade Actin FSL/FS APTT (green). Results are presented as percentages, with 95% confidence intervals provided in parentheses.

Eight samples (6%) were identified as positive for LA in at least two of the three assays and were classified as true positives. The PTT‐LA/Staclot LA results were positive in 8 samples (6%) and negative in 128 samples (94%), with a sensitivity of 100% (95% CI: 63.1–100) and a specificity of 100% (95% CI: 97.2–100). HemosIL SCT results were positive in 25 samples (18%) and negative in 111 samples (82%), with a sensitivity of 100% (95% CI: 63.1–100) and a specificity of 87% (95% CI: 79.6–92.1). Finally, Dade Actin FSL/FS APTT results were positive in 9 samples (7%) and negative in 127 samples (93%), with a sensitivity of 87.5% (95% CI: 47.3–99.7) and a specificity of 98% (95% CI: 94.5–99.8). The results are illustrated in Figure 1.

### 3.3 Reclassification of Results Based on the Modified HemosIL Silica Clotting Time Protocol

Of the 136 samples initially analyzed in the study, 124 were re‐tested using a modified HemosIL SCT protocol, which featured an increased concentration of phospholipids in the screening reagent. Among the 13 samples that were positive in the original HemosIL SCT assay, five (38%) remained positive following the assay modification. Notably, seven of the eight samples that became negative by the modified HemosIL SCT reagent had already tested negative on the other two platforms.

The agreement between the modified HemosIL SCT and Dade Actin FSL/FS APTT was substantial, with agreement in 121 samples (98%), comprising four positive and 117 negative (*k* = 0.71). The agreement between the modified HemosIL SCT and PTT‐LA/Staclot LA was also substantial, with 122 (98%) concordant results, of which four were positive and 118 negative (*k* = 0.79).

**FIGURE 2:**
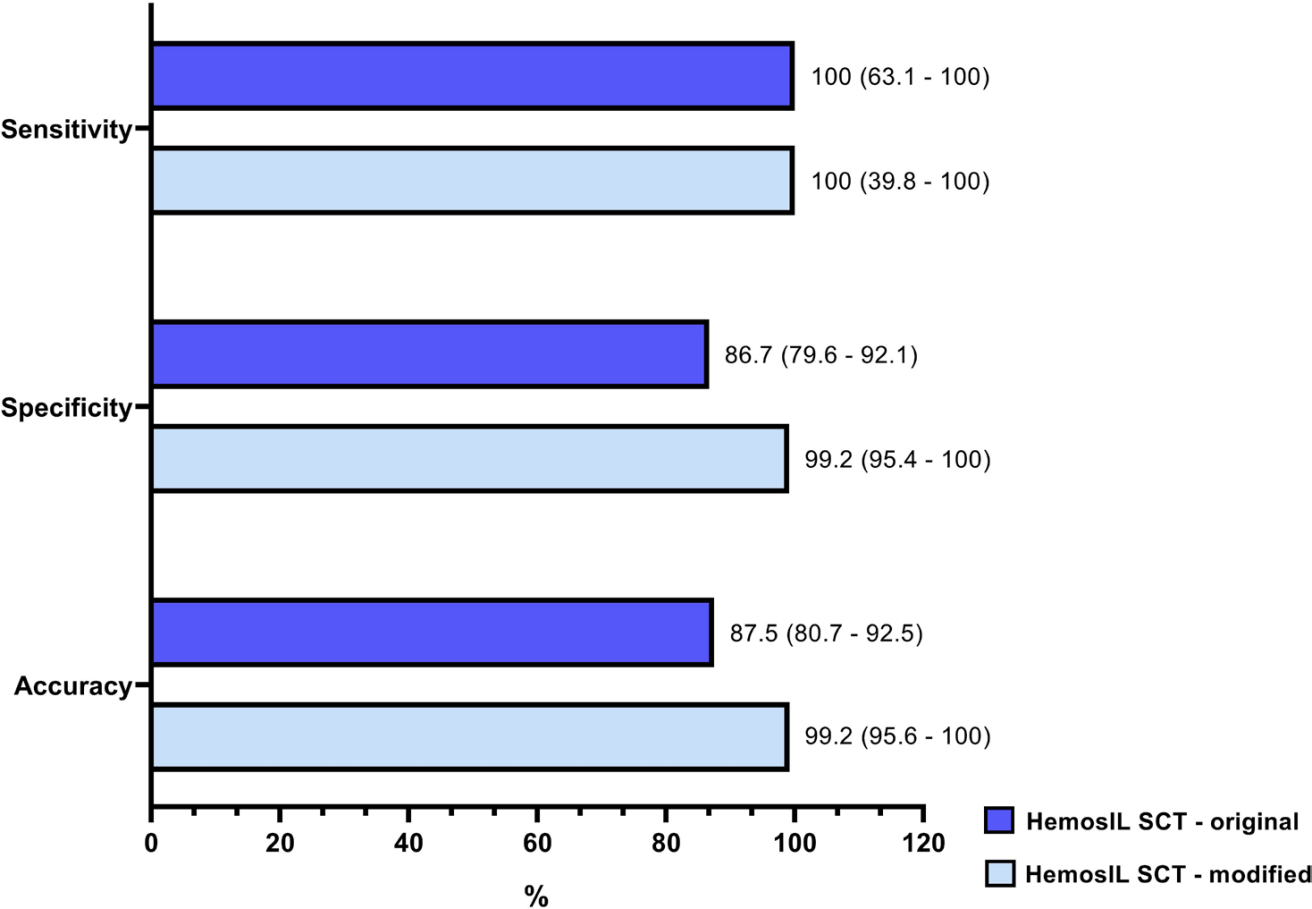
Sensitivity, specificity and accuracy after the modification in the HemosIL SCT. This figure illustrates comparison of the sensitivity, specificity, and accuracy of the original and modified HemosIL SCT assay for the detection of lupus anticoagulant. Original HemosIL SCT (dark blue) and modified HemosIL SCT (light blue) are evaluated. Results are presented as percentages, with 95% confidence intervals shown in parentheses.

The modified HemosIL SCT assay was positive in five samples (4%) and negative in 119 samples (96%), achieving a sensitivity of 100% (95% CI: 39.8–100) and an improved specificity of 99% (95% CI: 95.4–100). These results are illustrated in Figure 2.

## 4 Discussion

In this study, we demonstrated that the results agreement between Dade Actin FSL/FS APTT and PTT‐LA/Staclot LA assays is substantial, while HemosIL SCT assay yields the most discrepant results, particularly driven by a high rate of positivity. Moreover, our findings indicate that PTT/Staclot LA exhibits the highest specificity among the three assays. In contrast, HemosIL SCT assay, despite demonstrating good sensitivity (100%), exhibits the lowest specificity (87%) among the three. Dade Actin FSL/FS APTT has good specificity (98%), but moderate sensitivity (87%).

The accurate diagnosis and interpretation of LA results represent a significant challenge, primarily due to the absence of standardization or harmonization of assay and methodologies [14]. Furthermore, comparative studies of the available APTT‐LA assays are scarce. In the 90's, a study by Denis‐Magdelaine et col. demonstrated that reagents containing phospholipids derived from animal brains were the most sensitive and responsive compared to those based on human or soy‐derived phospholipids. To date, more studies are needed to ensure the accuracy and reliability of LA diagnostics [15].

The 2020 SSC‐ISTH guidelines recommend silica as the preferred activator for the contact phase in APTT assays, over ellagic acid [7]. The guidelines also address the source of phospholipids used in LA assays, recommending an increased phospholipid concentration (whether bilayer or hexagonal) in the confirmatory step [7]. In this context, HemosIL SCT is the only assay tested that fully complies with the 2020 SSC‐ISTH guidelines, employing silica as the activator and using the same phospholipid source in both the screening and confirmatory steps. Although the guidelines recommend silica as the preferred activator and the use of the same phospholipid source across both steps, our study did not demonstrate a clear performance advantage for these parameters, as both the PTT‐LA/Staclot LA and Dade Actin FSL/FS assays also showed good performance.

Our findings demonstrated that the Dade Actin FSL/FS APTT assay, which uses ellagic acid, showed substantial agreement with the PTT‐LA/Staclot LA assay, which uses silica. These results suggest that both silica and ellagic acid can be effective activators for LA diagnosing and are aligned with previous studies demonstrating that the sensitivity of the APTT‐LA assays depends on the concentration of the phospholipid, not on the type of activator [11].

We hypothesized that the lower specificity of the HemosIL SCT assay was due to a low concentration of phospholipids in the screening phase. By increasing the phospholipid concentration in that step, the specificity of the HemosIL SCT assay reagent was significantly improved, as well as the inter‐assay agreement. Therefore, our results suggest that the accuracy of APTT‐LA assays is influenced not only by the type of activator or the concentration of phospholipids used, but also by the combination of phospholipid concentration and structural characteristics (e.g., source—rabbit, soy, synthetic—and configuration—hexagonal vs. bilayer).

Lack of specificity is a critical issue in APS diagnosis, as patients are subjected to lifelong treatment [16]. Current efforts focus on enhancing diagnostic specificity in clinical practice [17], and the same emphasis should be placed on laboratory diagnostics. A recent study demonstrated that silica clotting time assays are less stable than dRVVT assays for LA detection [18]. Here, we have shown that results are highly dependent on the combination of activators, phospholipid type, and concentration. In this context, due to the variability across assays, an isolated positive APTT‐LA should be interpreted with caution, especially if the patient does not meet the clinical criteria for APS.

Some limitations of our study need to be pointed out. First, a significant number of samples in our study had missing clinical data, and additional diagnostic assays for APS were not performed. Although access to dRVVT results would provide additional information on LA positivity, it is important to note that this study focused on an analytical comparison of APTT‐LA assays performance rather than on LA detection itself. Nevertheless, future studies could explore the clinical relevance of single positivity for APTT‐LA, as well as the extent of discrepancies between APTT‐LA and dRVVT results. Second, some samples were obtained from individuals using anticoagulants (e.g., vitamin K antagonists, direct oral anticoagulants, or unfractionated heparin), which could potentially lead to both false‐positive and false‐negative results. Although samples were not screened for the presence of anticoagulants, it is important to note that we tested triplicates of the same sample and compared the results within these triplicates rather than between different samples. All samples analyzed across the three assays were subjected to the same pre‐analytical conditions, including the use of anticoagulants, clinical conditions, and blood sampling. In this context, the differences observed among the three assays are primarily attributable to analytical variables. Third, we defined a true positive sample as one that tested positive in at least two of the three assays evaluated in this study. While it could be argued that samples testing positive by HemosIL SCT should be considered true positives, given that this is the only assay that fully complies with the 2020 SSC‐ISTH guidelines, there is currently no strong evidence that alternative strategies are less accurate, and a gold standard for the APTT‐LA assay is still lacking.

## 5 Conclusion

This study demonstrated good agreement between the PTT‐LA/Staclot LA and the Dade Actin FSL/FS assays, as well as fair to moderate agreement with the HemosIL SCT assay. The performance of the HemosIL SCT assay improved with increasing phospholipid concentration. Our findings suggest that assays employing different activators and phospholipid sources than those preferred by the 2020 SSC‐ISTH guidelines can also achieve satisfactory performance in detecting AL.

## Author Contributions

Conceptualization: Bárbara G. Barion, Fernanda A. Orsi, Tania R. F. da Rocha. Investigation: Bárbara G. Barion, Maria Luiza S. A. de Paula, Thaís S. Saraiva, Bianca Stefanello, Vanderson Rocha, Paula R. Villaça, Fernanda A. Orsi, Tania R. F. da Rocha. Formal analysis: Bárbara G. Barion, Fernanda A. Orsi, Tania R. F. da Rocha. Resources: Vanderson Rocha, Fernanda A. Orsi, Writing: Bárbara G. Barion, Fernanda A. Orsi, Tania R. F. da Rocha. Final approval: Fernanda A. Orsi, Tania R. F. da Rocha.

## Conflicts of Interest

The authors declare no conflicts of interest.

## Supporting information


**Table S1:** Detailed sample results obtained using the modified HemosIL SCT.

## Data Availability

The data that support the findings of this study are available from the corresponding author upon reasonable request.
