# Supplementary material for: Laboratory Identification of Lupus Anticoagulant (LA) Using Different Activated Partial Thromboplastin Time (APTT) Assays
Source: Int J Lab Hematol. 2025 Aug 27;48(1):172–8. doi: 10.1111/ijlh.14549 (PMC12809375; doi:10.1111/ijlh.14549)
Supplement: Supplementary file 1 — Table S1: Detailed sample results obtained using the modified HemosIL SCT. [file IJLH-48-172-s001.docx]

**SUPPLEMENTARY MATERIAL**

**Supplementary Table 1** - Detailed sample results obtained using the modified HemosIL SCT.

| **Sample** | **Screen (s)** | **Screen ratio** | **Confirm (s)** | **Confirm ratio** | **Normalized ratio** |
| --- | --- | --- | --- | --- | --- |
| 1 | 27,3 | 0,78 | 32,5 | 0,9 | 0,86 |
| 2 | 34,1 | 0,97 | 27,6 | 1,04 | 0,94 |
| 3 | 34,9 | 0,99 | 37,5 | 1,04 | 0,96 |
| 4 | 34,3 | 0,97 | 31,6 | 0,87 | 1,12 |
| 5 | 37 | 1,05 | 33,6 | 0,93 | 1,13 |
| 6 | 36,5 | 1,04 | 33,9 | 0,94 | 1,11 |
| 7 | 35,5 | 1,01 | 33,1 | 0,91 | 1,1 |
| 8 | 36,2 | 1,03 | 37,5 | 1,04 | 0,99 |
| 9 | 32,2 | 0,91 | 31,8 | 0,88 | 1,04 |
| 10 | 39,4 | 1,12 | 35,7 | 0,99 | 1,13 |
| 11 | 38,5 | 1,09 | 40,2 | 1,11 | 0,98 |
| 12 | 39,5 | 1,12 | 36,2 | 1 | 1,12 |
| 13 | 37,1 | 1,05 | 29,5 | 0,81 | 1,29 |
| 14 | 39,1 | 1,11 | 30,9 | 0,85 | 1,3 |
| 15 | 36,7 | 1,04 | 38 | 1,05 | 0,99 |
| 16 | 36,8 | 1,05 | 32,3 | 0,89 | 1,17 |
| 17 | 36,3 | 1,03 | 30,1 | 0,83 | 1,24 |
| 18 | 40,8 | 1,16 | 36,8 | 1,02 | 1,14 |
| 19 | 37,8 | 1,07 | 32,1 | 0,89 | 1,21 |
| 20 | 42,1 | 1,2 | 38,6 | 1,07 | 1,12 |
| 21 | 35,4 | 1,01 | 29,9 | 0,83 | 1,22 |
| 22 | 38,7 | 1,1 | 35,5 | 0,98 | 1,12 |
| 23 | 37,4 | 1,06 | 33,8 | 0,93 | 1,14 |
| 24 | 30,7 | 0,86 | 36,9 | 0,9 | 0,96 |
| 25 | 32,7 | 0,92 | 37 | 0,9 | 1,02 |
| 26 | 43,3 | 1,22 | 43,1 | 1,05 | 1,16 |
| 27 | 48,4 | 1,36 | 48,7 | 1,19 | 1,15 |
| 28 | 38,1 | 1,07 | 36,3 | 0,89 | 1,21 |
| 29 | 41 | 1,15 | 42,1 | 1,03 | 1,12 |
| 30 | 33,1 | 0,93 | 36,8 | 0,9 | 1,04 |
| 31 | 42,8 | 1,21 | 39,3 | 0,96 | 1,26 |
| 32 | 43 | 1,21 | 38,3 | 0,93 | 1,3 |
| 33 | 31,3 | 0,88 | 33,9 | 0,83 | 1,07 |
| 34 | 32,2 | 0,91 | 32,3 | 0,79 | 1,15 |
| 35 | 39,9 | 1,12 | 44,8 | 1,09 | 1,03 |
| 36 | 33,2 | 0,94 | 30,6 | 0,765 | 1,25 |
| 37 | 37,4 | 1,05 | 35 | 0,85 | 1,23 |
| 38 | 36,6 | 1,03 | 35,8 | 0,87 | 1,18 |
| 39 | 39,7 | 1,12 | 37,5 | 0,91 | 1,22 |
| 40 | 38 | 1,07 | 37,7 | 0,92 | 1,16 |
| 41 | 32,2 | 0,91 | 33 | 0,8 | 1,13 |
| 42 | 32,5 | 0,92 | 38,6 | 0,94 | 0,97 |
| 43 | 37,6 | 1,06 | 37,9 | 0,92 | 1,15 |
| 44 | 32,6 | 0,92 | 29,5 | 0,72 | 1,28 |
| 45 | 31,2 | 0,88 | 31,9 | 0,78 | 1,13 |
| 46 | 29,3 | 0,83 | 30,8 | 0,75 | 1,1 |
| 47 | 36 | 1,01 | 37,8 | 0,92 | 1,1 |
| 48 | 43,9 | 1,24 | 37,6 | 0,92 | 1,35 |
| 49 | 36,4 | 1,03 | 36,9 | 0,9 | 1,14 |
| 50 | 36,1 | 1,02 | 36,8 | 0,9 | 1,13 |
| 51 | 37,5 | 1,06 | 29,3 | 0,71 | 1,48 |
| 52 | 34,4 | 0,97 | 33,4 | 0,81 | 1,19 |
| 53 | 36 | 1,01 | 38,1 | 0,93 | 1,09 |
| 54 | 35,8 | 1,01 | 34,4 | 0,84 | 1,2 |
| 55 | 35 | 0,99 | 32,2 | 0,79 | 1,26 |
| 56 | 33,7 | 0,95 | 34,3 | 0,84 | 1,13 |
| 57 | 35,6 | 1 | 35,9 | 0,88 | 1,15 |
| 58 | 31,1 | 0,88 | 29,8 | 0,73 | 1,21 |
| 59 | 36,3 | 1,02 | 35,5 | 0,87 | 1,18 |
| 60 | 31,7 | 0,89 | 34,3 | 0,84 | 1,07 |
| 61 | 40,2 | 1,13 | 39,4 | 0,96 | 1,18 |
| 62 | 33,6 | 0,95 | 29,3 | 0,71 | 1,32 |
| 63 | 36,1 | 1,02 | 40 | 0,98 | 1,04 |
| 64 | 35,5 | 1 | 29,8 | 0,73 | 1,38 |
| 65 | 34,1 | 0,96 | 36,2 | 0,88 | 1,09 |
| 66 | 36 | 1,01 | 33,6 | 0,82 | 1,24 |
| 67 | 41,3 | 1,16 | 34,6 | 0,84 | 1,38 |
| 68 | 41,6 | 1,17 | 32 | 0,78 | 1,5 |
| 69 | 35,2 | 0,99 | 32,5 | 0,79 | 1,25 |
| 70 | 33 | 0,93 | 31,74 | 0,77 | 1,2 |
| 71 | 40,9 | 1,15 | 40,1 | 0,98 | 1,18 |
| 72 | 41 | 1,15 | 36,6 | 0,89 | 1,29 |
| 73 | 38,7 | 1,09 | 38,5 | 0,94 | 1,16 |
| 74 | 39,1 | 1,1 | 39,1 | 0,95 | 1,15 |
| 75 | 34,1 | 0,96 | 30,8 | 0,75 | 1,28 |
| 76 | 40,5 | 1,14 | 35,8 | 0,87 | 1,31 |
| 77 | 34,1 | 0,96 | 32,2 | 0,79 | 1,22 |
| 78 | 41,8 | 1,18 | 43,8 | 1,07 | 1,1 |
| 79 | 29,6 | 0,83 | 32,9 | 0,8 | 1,03 |
| 80 | 42,7 | 1,2 | 38,6 | 0,94 | 1,28 |
| 81 | 40,5 | 1,14 | 35,7 | 0,87 | 1,31 |
| 82 | 32,1 | 0,9 | 28,3 | 0,69 | 1,31 |
| 83 | 38,4 | 1,08 | 35 | 0,85 | 1,27 |
| 84 | 36,3 | 1,02 | 32 | 0,78 | 1,31 |
| 85 | 42,7 | 1,21 | 35,2 | 0,97 | 1,25 |
| 86 | 46,4 | 1,32 | 38,8 | 1,07 | 1,23 |
| 87 | 36,9 | 1,05 | 29,1 | 0,8 | 1,3 |
| 88 | 34 | 0,97 | 28,7 | 0,79 | 1,23 |
| 89 | 44,3 | 1,25 | 35,3 | 0,86 | 1,45 |
| 90 | 36,8 | 1,04 | 32,6 | 0,79 | 1,32 |
| 91 | 43,3 | 1,23 | 40,1 | 0,98 | 1,25 |
| 92 | 44,8 | 1,27 | 37,3 | 0,91 | 1,39 |
| 93 | 39,7 | 1,12 | 37,9 | 0,92 | 1,22 |
| 94 | 39,6 | 1,12 | 37,2 | 0,9 | 1,24 |
| 95 | 44,5 | 1,26 | 42,3 | 1,03 | 1,22 |
| 96 | 41 | 1,16 | 36 | 0,88 | 1,32 |
| 97 | 34,5 | 0,98 | 32,9 | 0,80 | 1,22 |
| 98 | 38,7 | 1,1 | 35,40 | 0,86 | 1,28 |
| 99 | 44,6 | 1,26 | 38,6 | 0,94 | 1,34 |
| 100 | 36,9 | 1,04 | 31,5 | 0,77 | 1,35 |
| 101 | 40,4 | 1,14 | 38,9 | 0,95 | 1,2 |
| 102 | 41,7 | 1,18 | 35,6 | 0,87 | 1,35 |
| 103 | 44 | 1,25 | 39,2 | 0,95 | 1,31 |
| 104 | 39,3 | 1,11 | 39,5 | 0,96 | 1,16 |
| 105 | 28,4 | 0,8 | 35,2 | 0,86 | 0,93 |
| 106 | 43,1 | 1,22 | 44,7 | 1,09 | 1,12 |
| 107 | 46,2 | 1,31 | 47,9 | 1,17 | 1,2 |
| 108 | 44,4 | 1,26 | 47,2 | 1,15 | 1,09 |
| 109 | 34,2 | 0,97 | 35 | 0,85 | 1,13 |
| 110 | 34,5 | 0,98 | 34,1 | 0,83 | 1,18 |
| 111 | 30,8 | 0,87 | 32,7 | 0,8 | 1,09 |
| 112 | 33,6 | 0,95 | 31,3 | 0,76 | 1,25 |
| 113 | 36,4 | 1,03 | 45,5 | 1,11 | 0,93 |
| 114 | 36,6 | 1,04 | 38,1 | 0,93 | 1,12 |
| 115 | 38,4 | 1,09 | 39,8 | 0,97 | 1,12 |
| 116 | 29 | 0,82 | 32,3 | 0,79 | 1,04 |
| 117 | 37 | 1,05 | 39,9 | 0,97 | 1,08 |
| 118 | 34,4 | 0,97 | 48,2 | 1,18 | 0,83 |
| 119 | 36,4 | 1,03 | 44,1 | 1,08 | 0,96 |
| 120 | 38 | 1,08 | 42,4 | 1,03 | 1,04 |
| 121 | 36,4 | 1,03 | 39,7 | 0,97 | 1,06 |
| 122 | 36,2 | 1,03 | 37,7 | 0,92 | 1,12 |
| 123 | 38,3 | 1,08 | 39 | 0,95 | 1,14 |
| 124 | 37,5 | 1,06 | 37 | 0,9 | 1,18 |
| 125 | 30 | 0,85 | 32,1 | 0,78 | 1,09 |
| 126 | 33,3 | 0,94 | 32 | 0,78 | 1,21 |
| 127 | 33,5 | 0,95 | 30,8 | 0,75 | 1,26 |
| 128 | 37,1 | 1,05 | 43,3 | 1,06 | 1 |
| 129 | 42,9 | 1,22 | 40,1 | 0,98 | 1,24 |
| 130 | 33,1 | 0,94 | 49,1 | 1,2 | 0,78 |
| 131 | 33,7 | 0,95 | 34,1 | 0,93 | 1,15 |
| 132 | 30,9 | 0,88 | 33,2 | 0,81 | 1,08 |
| 133 | 41,5 | 1,18 | 59,1 | 1,44 | 0,82 |
| 134 | 31,9 | 0,9 | 33,4 | 0,81 | 1,11 |
| 135 | 42 | 1,19 | 48 | 1,17 | 1,02 |
| **P99** | 46,33 | **1,32** | 48,96 | 1,20 | **1,47** |

**Note:** Results of 135 samples analyzed using the modified HemosIL SCT.

**Abbreviations:** P99 = 99th percentile.
